# Enhancing pancreatic cancer treatment: the role of H101 oncolytic virus in irreversible electroporation

**DOI:** 10.3389/fimmu.2025.1546242

**Published:** 2025-03-18

**Authors:** Pu Xi, Dejun Zeng, Miao Chen, Lingmin Jiang, Yu Zhang, Dailei Qin, Zehui Yao, Chaobin He

**Affiliations:** ^1^ Department of Pancreatobiliary Surgery, State Key Laboratory of Oncology in South China, Guangdong Provincial Clinical Research Center for Cancer, Collaborative Innovation Center for Cancer Medicine, Sun Yat-sen University Cancer Center, Guangzhou, Guangdong, China; ^2^ Department of General Surgery, Pingshan District Central Hospital of Shenzhen, Shenzhen, China; ^3^ Department of Nuclear Medicine, The Central Hospital of Wuhan, Tongji Medical College, Huazhong University of Science and Technology, Wuhan, China; ^4^ State Key Laboratory of Ophthalmology, Zhongshan Ophthalmic Center, Sun Yat-sen University, Guangzhou, China

**Keywords:** irreversible electroporation (IRE), reversible electroporation(RE), H101 oncolytic virus, JNK-MAPK, apoptosis

## Abstract

**Background:**

Irreversible Electroporation (IRE) offers a promising treatment for pancreatic cancer by using high-voltage pulses to kill tumor cells. But variations in tumor size and shape can lead to uneven electric fields, causing some cells to undergo only reversible electroporation (RE) and survive. However, RE can temporarily increase the permeability of the cell membrane, allowing small molecules to enter. H101 virus is an oncolytic adenovirus with deleted E1B-55kD and E3 regions that selectively targets and kills tumor cells. This study aimed to investigate whether the H101 oncolytic virus can serve as a supplementary therapeutic approach to kill tumors combined with RE.

**Methods:**

We first explored how RE and the H101 oncolytic virus, both individually and together, affected tumor cell proliferation and migration in cellular experiments. Subsequent *in vitro* studies further assessed the effects of different treatments on tumor growth. To understand the mechanisms of pathway changes in tumors from different treatment groups, we analyzed tumor samples from each group using bulk RNA sequencing (bulk RNA-seq) and single-cell RNA sequencing (scRNA-seq). Additional biochemical techniques were used to validate key molecular changes.

**Results:**

The combination of RE with the H101 oncolytic virus effectively inhibited pancreatic cancer cell proliferation and migration. Experiments using mouse subcutaneous tumor models confirmed that the combination therapy significantly reduced tumor growth. Further analysis bulk RNA-seq and scRNA-seq revealed that this combined approach activates the JNK-MAPK pathway, inducing apoptosis and enhancing therapeutic effects.

**Conclusions:**

This combination boosts therapeutic effectiveness by activating the JNK-MAPK pathway and promoting tumor cell apoptosis. These findings suggest that the H101 oncolytic virus could serve as a valuable adjunct to improve the efficacy of IRE treatment.

## 1 Introduction

Pancreatic cancer (PC) is a highly aggressive gastrointestinal malignancy, ranking ninth in global incidence but fourth in mortality (1). Surgery remains the only curative option for PC, yet only about 20% of patients are eligible for surgical intervention at the time of diagnosis (2). Additionally, approximately 40% of cases are diagnosed as locally advanced pancreatic cancer (LAPC), characterized by vascular invasion that precludes immediate surgical resection (3). For LAPC patients, the effectiveness of radiotherapy and chemotherapy is limited, and only a small subset qualify for conversion surgery. However, the success rates for such procedures are low, and postoperative complications are significant (4, 5). This underscores the urgent need for innovative treatment strategies.

Irreversible electroporation (IRE) is an emerging tumor ablation technique that differs from traditional thermal or cryoablation methods by not relying on temperature changes to destroy tumor cells. Instead, it uses high-voltage, short-duration electrical pulses to create irreversible nanopores in tumor cell membranes, altering their permeability and inducing cell death. IRE overcomes challenges such as heat sink effects and reduces thermal injury to adjacent structures, preserving vital blood vessels, bile ducts, and pancreatic ducts within the ablation zone (6). This results in fewer postoperative complications, including pancreatic and biliary fistulas and infections, thereby improving outcomes for LAPC patients (7, 8). However, the success of IRE depends on the precise application of electrical pulses, which can be influenced by tumor geometry, electrode positioning, and the electrical properties of surrounding tissues (9). Given the aggressive and irregular nature of pancreatic cancer, areas of insufficient field intensity within the tumor can result in incomplete ablation. This phenomenon, termed reversible electroporation (RE), may paradoxically stimulate tumor growth (10–12). According to a previous research, electric field intensities below 1000 V/cm predominantly induced RE rather than IRE (13). Consequently, combining IRE with supplementary therapies is essential to enhance its efficacy.

The H101 oncolytic virus, a recombinant virus selectively targeting cancer cells while sparing normal ones (14), has shown promise in treating advanced cancers such as nasopharyngeal carcinoma and melanoma (15, 16). The virus exerts its anticancer effects through multiple mechanisms, including direct infection and lysis (17), induction of immunogenic cell death (18), and targeting of tumor-associated fibroblasts and other stromal cells, thereby disrupting the complex tumor structure and promoting the infiltration of cytotoxic immune cells (19). Previous literature also indicated that IRE can reshape the tumor microenvironment by enhancing microvascular density and tumor vascular permeability (20). Additionally, the membrane pores created by IRE or RE can facilitate the entry of drugs and genes (21, 22), potentially increasing the local concentration of the H101 virus within tumors and eliminating residual cancer cells that survive after RE.

In this study, we found that RE alone did not induce cytotoxicity but instead enhanced cellular migration. However, combining RE with the H101 oncolytic virus demonstrated significantly improved tumor-killing effects and reduced pancreatic tumor migration compared to H101 virus treatment alone. These findings suggested that the H101 oncolytic virus is a potent complementary therapy, capable of enhancing the efficacy of IRE for the treatment of pancreatic cancer.

## 2 Materials and methods

### 2.1 Cell culture and viruses

The Bxpc-3 human pancreatic adenocarcinoma cell line and the Pan02 murine pancreatic adenocarcinoma cell line were obtained from the Cell Bank of the Chinese Academy of Sciences (Shanghai, China). Cells were maintained at 37°C in a 5% CO2 humidified incubator using DMEM (C11995500BT, Gibco) or RPMI 1640 (R8758, Gibco) media, both supplemented with 10% heat-inactivated fetal bovine serum (A5669701, Gibco) and 1% Penicillin-Streptomycin (15140122, ThermoFisher). The H101 oncolytic virus was generously donated by Shanghai Sunway Biotech (Shanghai, China).

### 2.2 Establishment of animal models

In this study, 6-week-old C57BL/6 female mice were used. All procedures involving animals were authorized by the Animal Care and Use Committee of Sun Yat-sen University (ID, L102012021009A). 1×10^6 Pan02 cells were implanted into the right flank of C57BL/6 mice. After a period of 2 weeks, palpable tumors with a diameter of approximately 7 to 8 mm were formed. The dimensions of the tumors, including length (A) and width (B), were assessed, and the tumor volume was determined using the formula: V = (A × B^2)/2. Upon reaching a tumor volume of 200-250 mm³, the mice were randomly assigned to four groups. The first group received intratumor injections of PBS three times a week, serving as a placebo. The second group received low-field electrical stimulation once a week. The third group received intratumor injections of H101 virus (1×10^8^ TCID50) three times a week. The fourth group received a combination of intratumor injections of H101 virus (three times a week) and low-field electrical stimulation (once a week). Tumors were treated with intratumoral virus injection immediately after exposure to low-field intensity stimulation. The dimensions of the tumors were recorded every 3 days until the sacrifice on day 35.

### 2.3 Electroporation

The *in vivo* and *in vitro* electroporation experimental methods are as described in our previous article (23). ECM 830 electroporator (BTX Harvard Apparatus, Holliston, MA) was used to generate electric pulses. For *in vitro* experiments, a 400-μL cell suspension of 1×10^6 cells, post-digestion and counting, was positioned in an electroporation cuvette (1652088; BTX, Holliston, MA, USA) between two 4-mm spaced aluminum electrodes. For *in vivo* experiments, electroporation was conducted using a 2-needle array electrode (BTX item #45–0168, BTX Harvard Apparatus, Holliston, MA) with a 5 mm gap. The electrode was inserted sequentially into the tumor along the X, Y, and Z axes to ensure the efficacy of electroporation. The parameters for electroporation were as follows: voltage: 150 V; pulse duration: 100 ms; pulse frequency: 1 Hz; pulse number: 80.

### 2.4 Cell viability assay

Cell Counting Kit-8 (CCK8) Assay Cell viability was analyzed using the cell counting kit-8 (CCK-8) assay (HYC500, HUAYUN). Triturated cells were seeded in 96-well flat-bottomed plates at a density of 2*103 in 100 μL of conditioned medium per well, and 10 μL of CCK-8solution was added to each well. After 2 h of incubation, the absorbance of each well at a wavelength of 450 nm was quantified.

### 2.5 Flat plate clone formation

Post-treatment, 500 cells were plated into each well of a 6-well plate and cultured for 14 days, with media changes occurring every 3 days. The cells were then fixed using a 4% formaldehyde solution and stained with crystal violet (E607309-0100, Sangon Biotech, Shanghai, China). Subsequently, the colonies were counted and subjected to analysis.

### 2.6 Cell scratch and transwell migration assay

For the scratch wound assay, the tumor cells of different treatment groups were counted as 1×10^5^ and then placed in 12 well plates. Subsequently, the cell layer was scratched with the tip of a 20 µL sterile pipette to create a wound gap. The migrated rate is quantified as the distance of wound closure at 0 and 24 hours relative to the initial wound length. For the transwell migration assay, 8 μm pore size transwell chambers (353097, Corning) were utilized. The lower chamber was supplemented with medium containing 20% FBS. After teatment, the cells were counted to 1×10^4 and placed separately in the upper chamber. After 24 hours, cancer cells that had penetrated and adhered to the underside of the filter were fixed with paraformaldehyde and stained with crystal violet. They were then imaged and counted under a 20X objective lens. The statistical analysis of migrating cell counts was based on data from three replicate experiments, with the average determined from assessments of five microscopic fields per experiment.

### 2.7 Western blotting

The protein lysates were generated using RIPA buffer, resolved by sodium dodecyl sulfate–polyacrylamide gel electrophoresis gel, moved to polyvinylidene difluoride membranes (Millipore, Billerica, MA, USA), and incubated with primary antibodies at 4°C overnight. The following antibodies were utilized: GAPDH (10494-1-AP, Proteintech), Snail (A11794, ABclonal), Vimentin (60330-1-Ig, Proteintech), E-cadherin (20874-1-AP, Proteintech), N-cadherin (22018-1-AP, Proteintech), Phospho-ERK1/2 (28733-1-AP, Proteintech), ERK (66192-1-Ig, Proteintech), Phospho -P38 (28796-1-AP, Proteintech), P38 (14064-1-AP, Proteintech), Phospho -JNK (80024-1-RR, Proteintech), JNK (24164-1-AP, Proteintech), Bc1-2 (60178-1-Ig, Proteintech), BAX (50599-2-Ig, Proteintech). After three washes with Tris-buffered saline containing 0.05% Tween-20, the membranes were incubated with either secondary anti-rabbit (AS003, ABclonal) or anti-mouse antibodies (SA00001-1, Proteintech) for 1 hour at room temperature. Images were detected by Tanon-5200 chemiluminescent imaging system (Tanon, Shanghai, China). The signals were detected using the ECL Advance reagent (P10300, New Cell & Molecular Biotech Co.,Ltd) and quantified with ImageLab software. The experiment was conducted in triplicate.

### 2.8 Immunohistochemistry assay

4-μm-thick tumor sections were deparaffinized in xylene, rehydrated through a graded series of ethanol, and incubated in 0.3% H2O2 in methanol for 30 minutes. After washing with PBS, the sections were probed with a monoclonal antibody (ab15580, Abcam) at 4°C overnight. After washing, the sections were incubated with biotinylated goat anti-rabbit IgG for 2 hours at room temperature. Immunostaining was then detected using a streptavidin-horseradish peroxidase conjugate and diaminobenzidine. Subsequently, the sections underwent counterstaining with hematoxylin.

### 2.9 Flow cytometry

Following the manufacturer’s guidelines, the Annexin V-FITC/PI apoptosis detection kit (GOONIE, Cat. No. 100-101) was employed to assess cellular apoptosis. Cells were collected at a concentration of 1×10^5 cells per tube and stained in duplicate with 5 μl of APC-Annexin V conjugate and 5 μl of propidium iodide solution (10 μg/ml), with the staining process conducted in the dark for a duration of 30 minutes. Subsequently, the cells were analyzed using the CytoFLEX S system (Beckman Coulter, USA) via flow cytometry. The percentage of apoptotic cells was referred to as the apoptosis rate.

### 2.10 Tissue dissociation and cell purification

The dissociation and purification process of the tissue was as previously described (24). In short, the tissue were transported on ice in DMEM containing 1 mM protease inhibitor (CW2200S, CWBIO) to maintain viability, washed three times with PBS, then minced on ice. To digest the tissues, we employed a dissociation enzyme mixture containing 1 mg/ml of Type VIII Collagenase (C2139, Sigma), 2 mg/ml of Dispase II (4942078001, Sigma), 1 mg/ml of Trypsin Inhibitor (T6522, Sigma-Aldrich), and 1 unit/ml of DNase I (M0303S, NEB), all dissolved in PBS supplemented with 5% FBS. After filtration through a 40 μm nylon cell strainer (model 352340, Falcon), the cell suspension underwent erythrocyte removal using red blood cell lysis buffer (1966634, Invitrogen). Subsequently, cells were stained with 0.4% trypan blue solution (T10282, Invitrogen) to discern cell viability. Finally, the cells were diluted in PBS containing 0.04% bovine serum albumin (BSA) to approximately 1 × 10^6^ cells per milliliter for single-cell sequencing applications.

### 2.11 10× library preparation and sequencing

Cells were prepared in accordance with the standardized protocol of the Chromium single-cell 3′ reagent kit, aiming to capture a range of 5000 to 10,000 cells per chip position (utilizing V2 chemistry). Subsequent steps, encompassing library preparation, were executed following the manufacturer’s established guidelines meticulously.

### 2.12 Single cell RNA-seq data processing

Illumina HiSeqXTen platforms were utilized for the sequencing of single-cell libraries, employing a paired-end approach with 150 nucleotide reads. The data obtained were then subjected to analysis via the Cell Ranger 2.1.0 software suite, adhering to the preset and advised settings. The resulting FASTQ files from the Illumina sequencing were mapped to the GRCm39 reference genome using the STAR alignment method (25). The processed output was subsequently loaded into Seurat version 5.0.0 to conduct quality control and further analysis on our scRNA-seq dataset. Unless otherwise stated, all operations were performed using the default settings. Cells of inferior quality, characterized by having fewer than 300 genes per cell, fewer than 5 cells per gene, or a mitochondrial gene expression exceeding 15%, were removed from the dataset.

### 2.13 Identification of cell types and subtypes by nonlinear dimensional reduction (umap)

The R-based Seurat package was utilized to discern primary cell types. A set of highly variable genes was derived and leveraged for conducting Principal Component Analysis (PCA). These clusters were then visualized through UMAP, utilizing the first ten principal components (PCs) previously computed via the RunUMAP function. To delineate the cellular identities within these clusters, we referenced the expression of established markers: Ptprc for immune cells, Krt8 and Krt19 for epicardial cells, Pecam1 for endothelial cells, and Acta2 for stromal cells (26). Cluster-defining marker genes were pinpointed by executing the FindAllMarkers function within the Seurat package, applied to the normalized gene expression dataset.

### 2.14 Bulk RNA-seq

In brief, we began with 500ng of purified RNA with a high integrity score (RIN >7), which was selectively targeted for polyadenylated mRNA. This was followed by a series of steps including fragmentation, synthesis of cDNA using random primers with the NEBNext protocol, indexing via PCR, and then selecting and measuring the cDNA fragments with KAPA reagents from Roche. The prepared cDNA libraries were then sequenced on the Illumina NovaSeq 6000 platform. The alignment to the GRCm39 reference genome and the counting of reads were executed using the previously outlined methods (26), employing the STAR aligner (version 2.7.8), featureCounts (version 1.6.4), and Ensembl gene transcripts (version 104). The analysis of differential gene expression was conducted using the DESeq2 software package (version 1.30) (27).

### 2.15 Biological signaling pathways analysis

For the biological pathways of differential genes, we conducted Go (Gene Ontology), KEGG (Kyoto Encyclopedia of Genes and Genomes), and GSEA (Gene Set Enrichment Analysis) analyses using the “clusterProfiler” (V.4.8.3) R package.

### 2.16 Statistical analysis

Statistical analyses were conducted utilizing GraphPad Prism version 8.00 software (GraphPad Software, La Jolla, CA, USA). Appropriate statistical tests, including Student’s t-test or analysis of variance (ANOVA), were selected to compare different groups in the *in vitro* study. Repeated-measures one-way ANOVA was employed to analyze tumor volume data. Standard deviations (SDs) were represented by error bars. Statistical significance was set at a P value of less than 0.05.

## 3 Results

### 3.1 Low-intensity electric fields enhance cell migration without causing cytotoxicity

**Figure 1:**
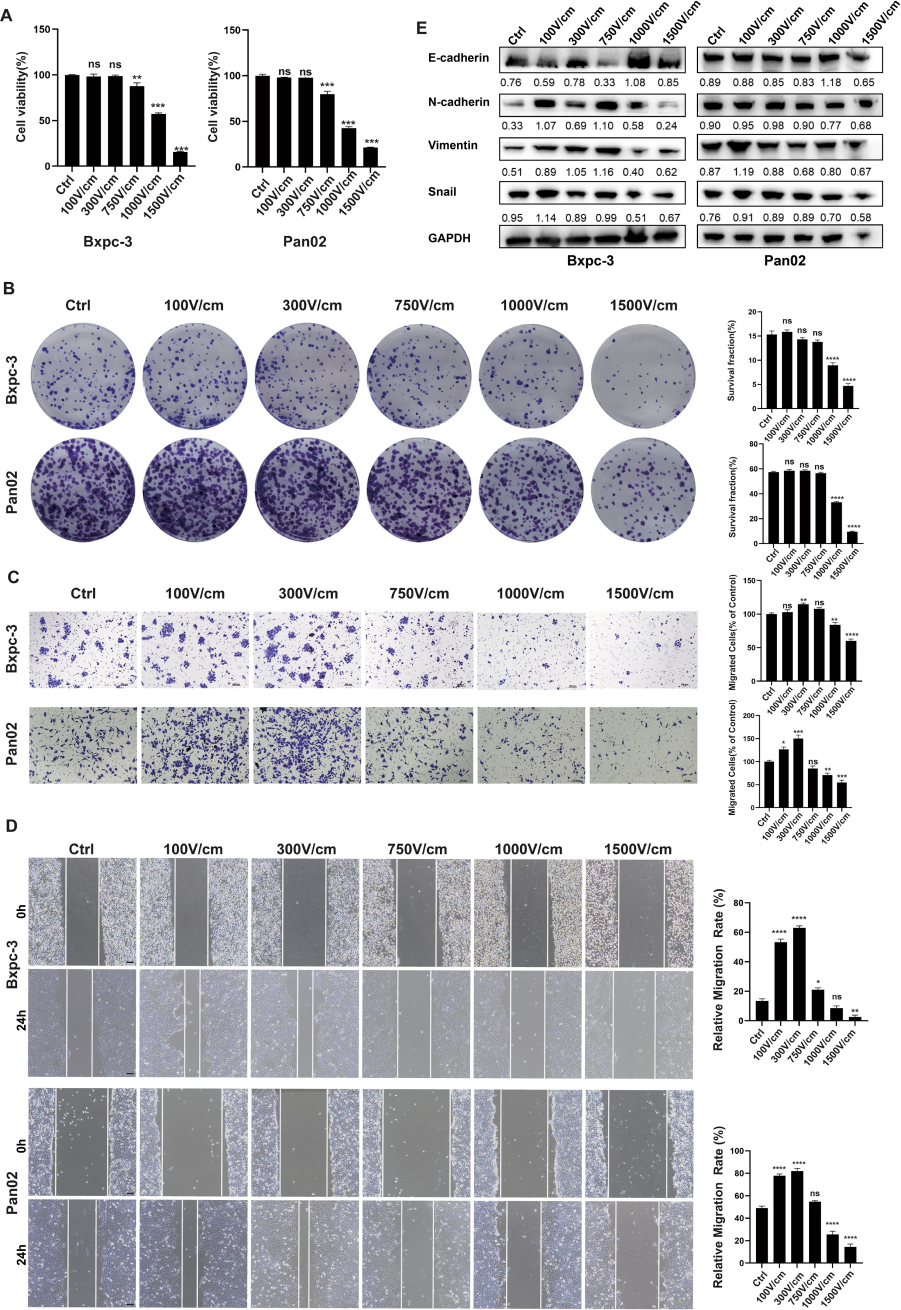
Low-Intensity Electric Fields Enhance Cell Migration Without Causing Cytotoxicity. **(A)**, The CCK-8 results for tumor cell survival rates after stimulation with different field strength groups, CCK-8 assay results showed that electric field intensities below 300 V/cm have no significant cytotoxic effect, while high-intensity fields (≥1000 V/cm) significantly reduce cell viability. **(B)**, Colony formation assay of cell viability under different field strengths, no significant cytotoxicity was observed with electric field intensities below 750 V/cm, while high-intensity fields (≥1000V/cm) significantly reduced colony formation. **(C, D)**, Representative images of the transwell migration assay and cell scratch assay (at 0h and 24h), low-intensity electric fields (≤300 V/cm) enhance cell migration, while high-intensity fields (≥1000 V/cm) inhibit migration, scale bar, 100 μm. **(E)**, Western blot analysis of EMT markers in tumor cells treated with different electric field intensities for 24 hours. Low-intensity fields (100–750 V/cm) activate the EMT pathway, as indicated by increased N-cadherin, Vimentin, and Snail expression and decreased E-cadherin expression. High-intensity fields (≥1000 V/cm) inhibited EMT, as shown by the opposite expression pattern. Ns, not significant; *p < 0.05; **p < 0.01; ***p < 0.001, ****p < 0.0001, significant difference compared with the control. Data are presented as mean ± SD.

We examined the effects of varying electric field intensities (100 V/cm to 1500 V/cm, including intermediate levels of 300 V/cm, 750 V/cm, and 1000 V/cm) on tumor cell viability and migration. Cells were exposed to 20 pulses, with each pulse lasting 100 microseconds at a frequency of 1 Hz. Cytotoxicity was assessed immediately using CCK8 and plate colony formation assays. Results showed no significant cytotoxicity below 750 V/cm, with most cells surviving intact (**Figures 1A, B**). Migration assays (scratch and transwell tests) demonstrated enhanced tumor cell migration at low-field intensities (<750 V/cm), while intensities above this threshold significantly inhibited migration (**Figures 1C, D**). Western blot analysis revealed that low-field stimulation activated epithelial-to-mesenchymal transition (EMT), as evidenced by increased expression of Snail, N-cadherin, and vimentin, along with decreased E-cadherin expression (**Figure 1E**).

### 3.2 Combined RE and H101 virus treatment suppresses tumor growth

**Figure 2:**
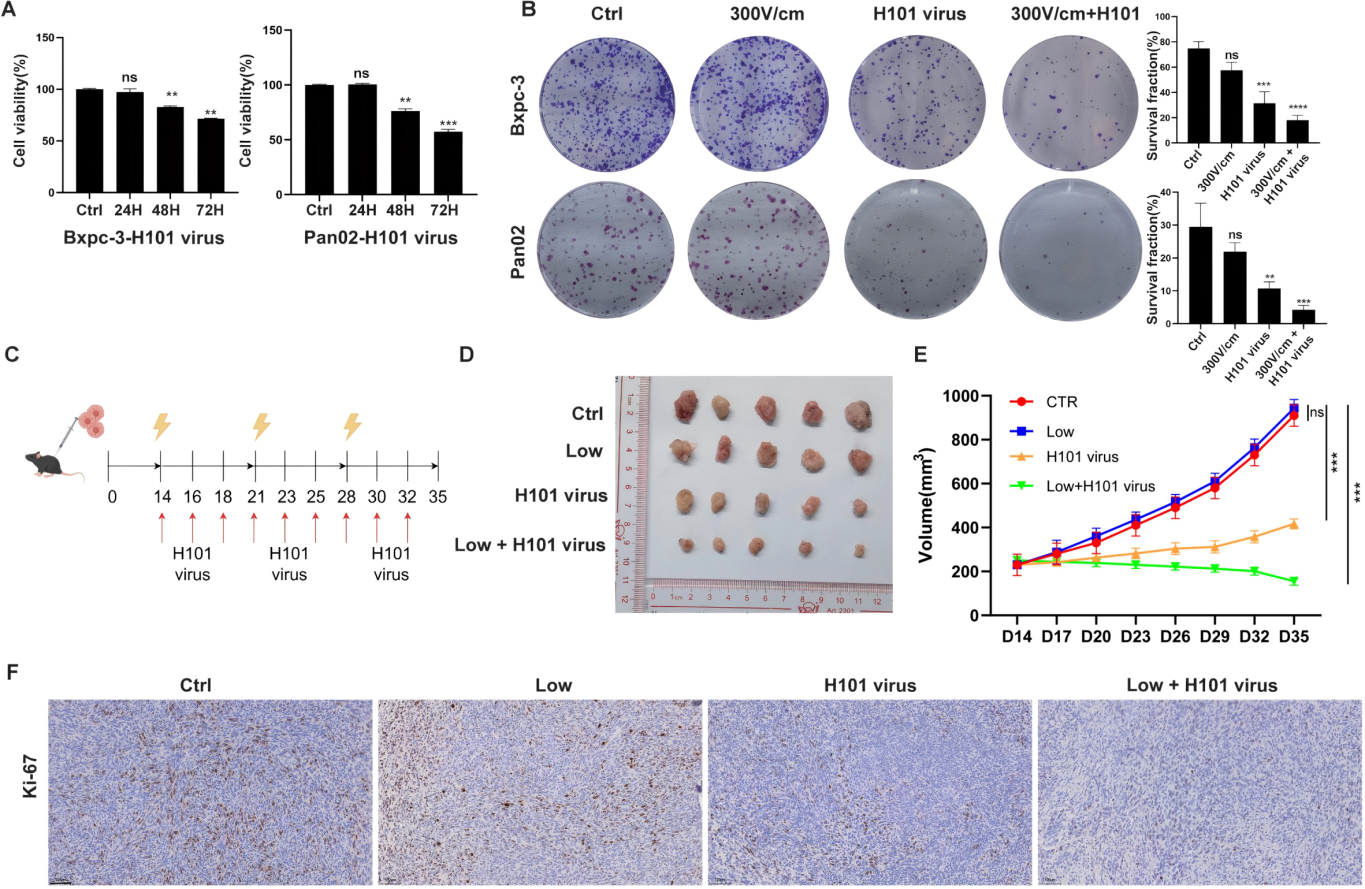
Combined RE and H101 Virus Treatment Suppresses Tumor Growth. **(A)**, The CCK-8 results for tumor cells after infection with a multiplicity of infection (MOI) of 100 at 24, 48, and 72 hours, a significant decrease in cell viability was observed. **(B)**, Colony formation assay of tumor cells after different treatment, the results showed that H101 virus alone reduces cell viability, and the combination of 300 V/cm electric field with H101 virus further enhanced the cytotoxic effect. **(C)**, The timeline and scheme of the experimental setup for subcutaneous animal experiments, 1×10^6 Pan02 cells were implanted into the right flank of C57BL/6 mice. Treatments included weekly low-field electrical stimulation and thrice-weekly intratumor injections of H101 virus (1×10^8 TCID50) over 35 days. Yellow lightning bolts denote electrical stimulation, and red arrows indicate virus injections. **(D)**, Representative images of tumors from each group at day 35. **(E)**, Tumor growth curve diagram, data represent mean ± SD for each group at the indicated time points. **(F)**, Representative Ki-67 immunohistochemicalstaining in subcutaneous tumor sections. Scale bar, 100 μm. Ns, not significant; **p < 0.01; ***p < 0.001, ****p < 0.0001,significant difference compared with the control.

To evaluate the H101 virus’s cytotoxic effects, tumor cells were infected with an MOI of 100, and cell viability was monitored at 24, 48, and 72 hours post-infection using CCK8 assays. The results demonstrated a time-dependent decline in cell viability following H101 virus infection (**Figure 2A**). Colony formation tests showed that the combined treatment with low-field intensity and H101 virus produced fewer and smaller colonies compared to treatments with low-field intensity or H101 virus alone (**Figure 2B**). *In vivo* experiments using mouse models further confirmed these findings, as tumor growth was significantly suppressed in the combination therapy group compared to individual treatments (**Figures 2C-E**). Immunohistochemical analysis of tumor tissues revealed reduced proliferation markers, such as Ki-67, in the combined treatment group (**Figure 2F**). These results confirm that the combination therapy effectively inhibits tumor growth both *in vitro* and *in vivo*.

### 3.3 Combined RE and H101 virus treatment inhibits tumor cell migration

**Figure 3:**
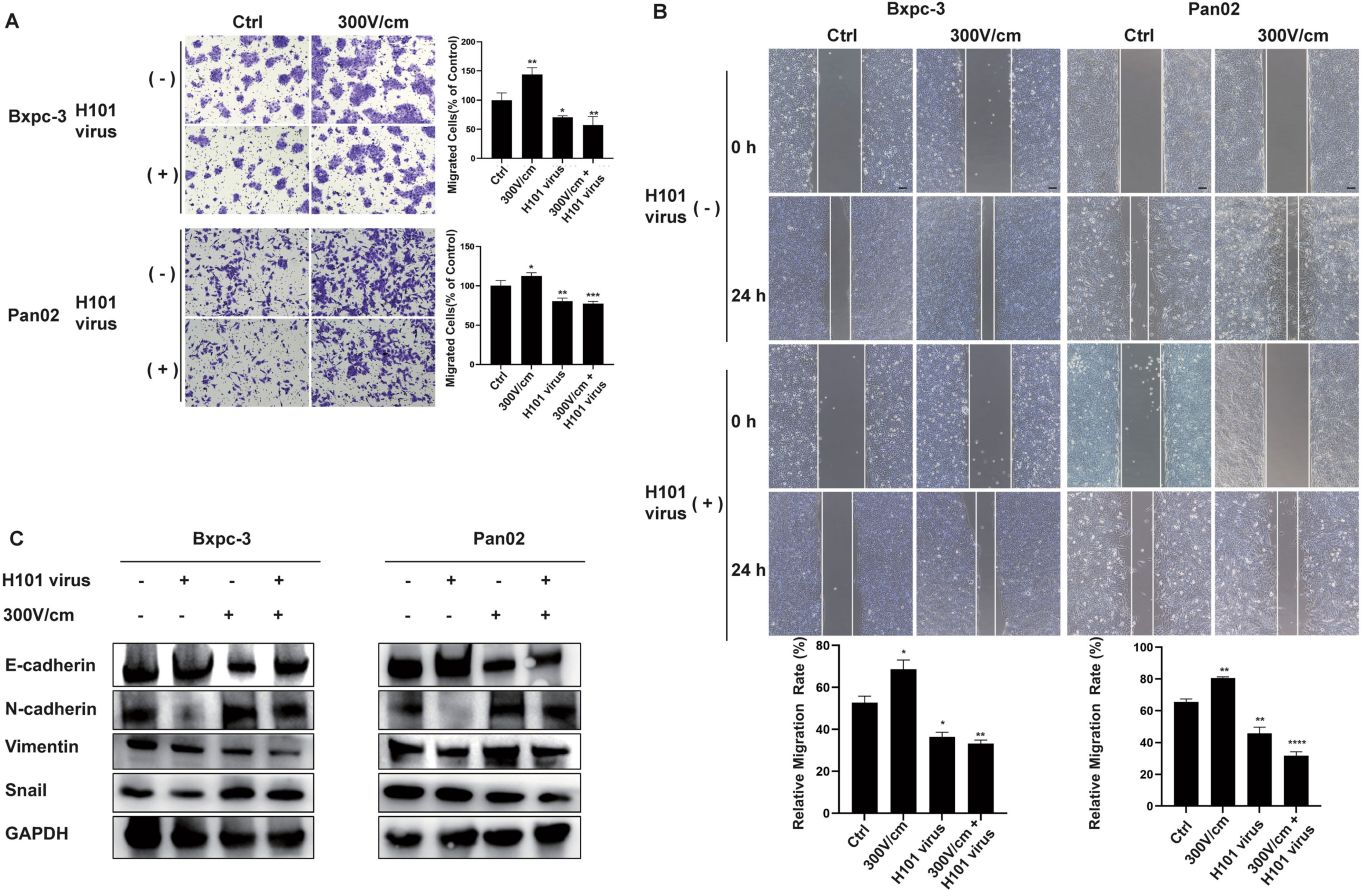
Combined RE and H101 Virus Treatment Inhibits Tumor Cell Migration. **(A, B)**,The low-intensity electric field (300 V/cm) enhanced tumor cell migration, while the addition of the H101 virus partially inhibited it, scale bar, 100 μm. Data are presented as mean ± SD. Ns, not significant; *p < 0.05; **p < 0.01; ***p < 0.001, ****p < 0.0001. **(C)** Western blot analysis of EMT-related protein levels.The low-intensity electric field stimulation promoted the EMT, while H101 virus partially reversed this effect.

To evaluate the impact of low-field intensity stimulation in combination with H101 virus on cellular migration, cell migration assays (scratch and transwell tests) were performed and showed that while low-field intensity stimulation initially enhanced tumor cell migration, subsequent treatment with H101 virus reversed this effect (**Figures 3A, B**). Western blot analysis indicated that the EMT process induced by low-field stimulation was mitigated by the H101 virus, as demonstrated by downregulation of Snail, N-cadherin, and vimentin, along with upregulation of E-cadherin (**Figure 3C**).

### 3.4 Bioinformatics analysis reveals MAPK pathway activation

**Figure 4:**
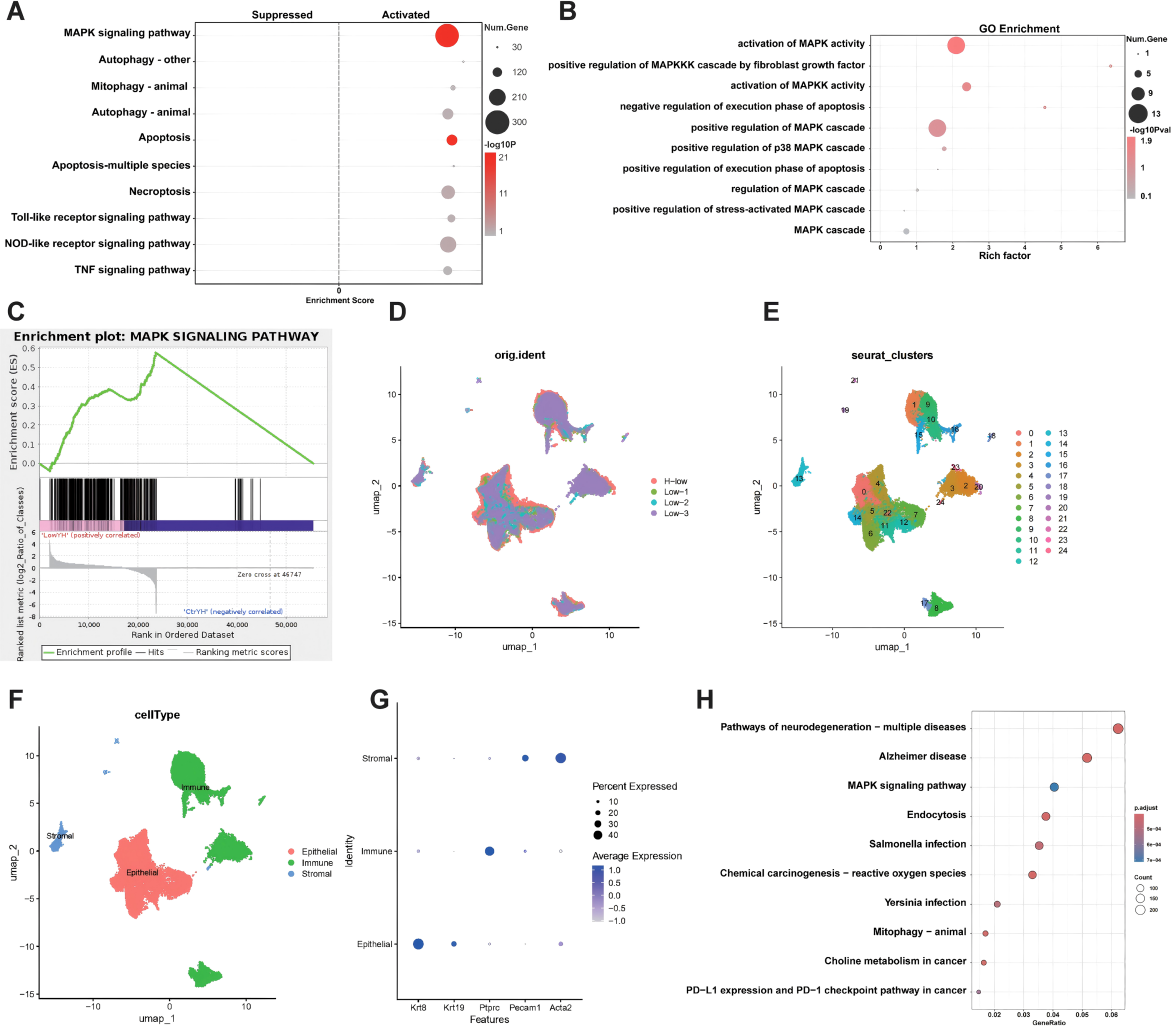
Bioinformatics Analysis Reveals MAPK Pathway Activation. **(A)**, The top 10 pathways activated by differential genes of bulk RNA-seq, the MAPK signaling pathway is significantly activated. **(B)**, GO enrichment analysis of MAPK-related biological processes. Key processes include the activation and regulation of the MAPK cascade, as well as apoptosis-related pathways. **(C)**, GSEA enrichment plot for the MAPK signaling pathway. The enrichment score curve indicates significant activation of the MAPK pathway. **(D-F)**, UMAP visualization of single-cell RNA sequencing data. **(D)** Cells are grouped by experimental conditions(Low means low-Intensity electric field. H-low means H101 virus plus low-Intensity electric field.) **(E)** Clusters of cells identified, labeled with cluster numbers. **(F)** Major cell types annotated, including epithelial, immune, and stromal populations. **(G)**, Dot plot of gene expression across different cell types. Expression of various marker genes (Krt18, Krt19, Ptprc, Pecam1, Acta2) is shown for stromal, immune, and epithelial cells. **(H)**, KEGG pathway enrichment analysis of scRNA-sequencing data. The MAPK signaling pathway is prominently enriched, ranked third in terms of gene ratio.

Tumor samples from mouse models treated with either low-field intensity alone or the combination therapy underwent bulk RNA-seq and scRNA-seq. Differentially expressed gene analysis from bulk RNA-seq indicated that the MAPK signaling pathway was the most significantly activated (**Figure 4A**). GO and GSEA corroborated this finding (**Figures 4B, C**). ScRNA-seq further detailed the pathway enrichment, with KEGG analysis confirming that the MAPK pathway was the primary enriched pathway across treatment groups (**Figures 4D-H**).

### 3.5 JNK-MAPK pathway activation induces apoptosis in tumor cells

**Figure 5:**
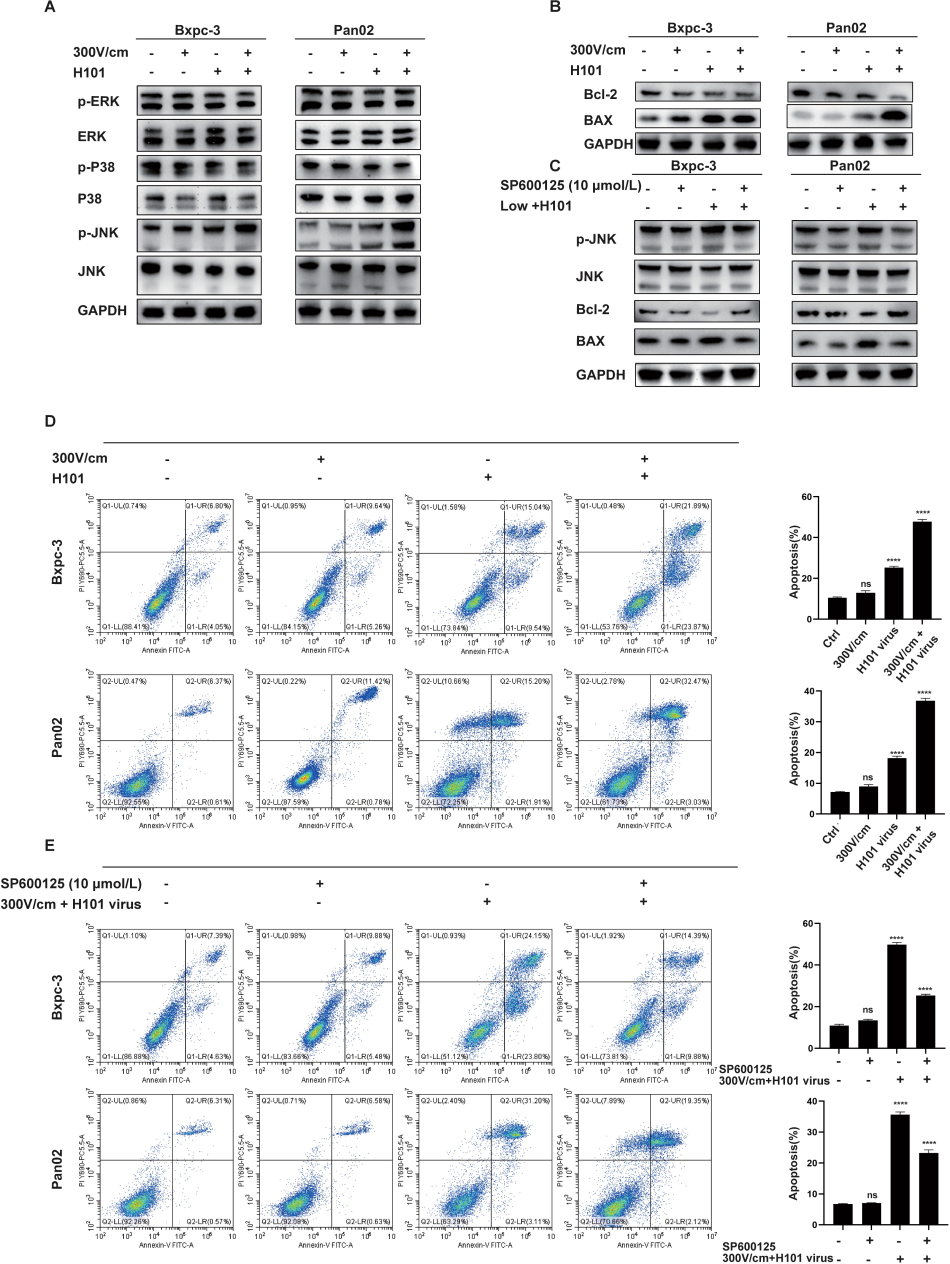
JNK-MAPK Pathway Activation Induces Apoptosis in Tumor Cells. **(A)**, Western blot analysis of MAPK-related protein levels. Low-intensity electric field (300 V/cm) combined with H101 virus treatment significantly activates the JNK-MAPK pathway, as indicated by increased p-JNK levels. No significant changes were observed in other MAPK pathway markers. **(B)**, Western blot analysis of apoptosis-related protein levels. The combined treatment significantly increased the expression of apoptotic markers (Bax). **(C)**, Western blot analysis of JNK-MAPK and apoptosis-related proteins. Treatment with JNK inhibitor (SP600125, 10 µmol/L) suppresses the JNK-MAPK and apoptosis pathways, as indicated by the reduced levels of p-JNK, and BAX. **(D)**, Flow cytometric analysis of apoptosis in tumor cells. Low intensity electric fields did not significantly increase the apoptotic cell population. The treatment with H101 virus alone or combined with low-intensity electric field stimulation enhanced apoptosis, with the highest rate observed in the combination group. **(E)**, Flow cytometric analysis of apoptosis after the addition of the JNK inhibitor.Treatment with the JNK inhibitor partially inhibits apoptosis in the combined treatment group. Data are presented as mean ± SD. Ns, not significant; ****p < 0.0001.

Based on the bioinformatics analysis results, we initially performed Western blot analysis to assess the expression levels of the three major MAPK subfamilies—JNK, ERK, and p38. It was showed that the combination therapy specifically enhanced JNK phosphorylation, with no significant effect on ERK or p38 expression (**Figure 5A**). The JNK signaling pathway, a key MAPK subfamily, plays a pivotal role in apoptosis. Flow cytometry and Western blot analyses demonstrated increased BAX expression, decreased BCL-2 expression, and a significantly higher apoptosis rate in the combination therapy group (**Figures 5B, D**). To explore JNK’s role in apoptosis, cells were pretreated with the JNK inhibitor SP600125. The inhibition of JNK reduced apoptosis, as shown by decreased BAX expression, increased BCL-2 expression, and lower apoptotic cell percentages (**Figures 5B, E**). These findings identify JNK as a critical mediator in apoptosis induction and highlight its role in the enhanced therapeutic efficacy of the combined low-field intensity and H101 virus treatment.

## 4 Discussion

In this study, we initially demonstrated that RE, while not significantly cytotoxic to tumor cells, unexpectedly enhanced their migratory ability. Conversely, the H101 oncolytic virus alone exhibited strong tumoricidal effects and reduced cell migration. When combined, the two approaches significantly enhanced tumor cell killing and migration inhibition. Bulk RNA-seq and scRNA-seq analyses revealed that the combination of RE and H101 virus activates the JNK-MAPK pathway, promoting tumor cell apoptosis and improving therapeutic efficacy compared to low-field stimulation alone.

Due to the invasion of adjacent structures or distant metastasis, only about 20% of patients with pancreatic cancer have the opportunity for radical resection at initial diagnosis (28). For patients with LAPC, which accounts for nearly 40% of all pancreatic cancer cases, the opportunity for radical surgical resection is also forfeited (29). IRE, as an emerging and effective treatment modality, has been demonstrated to significantly prolong the survival of patients with LAPC (8, 30, 31). However, due to tumor heterogeneity and variable electric field distribution within tumors, some areas may only experience RE, allowing tumor cells to survive and potentially leading to recurrence. As a new localized treatment, IRE can induce short and high-voltage current pulses to disrupt the integrity of cell membrane, causing cell apoptosis and death (32). In this study, electric fields below 750 V/cm were shown to cause RE without cytotoxicity. Furthermore, cell migration assays revealed enhanced tumor cell motility after low-field stimulation, likely due to EMT. These findings suggested that RE might contribute to recurrence after IRE treatment, highlighting the need for complementary therapies.

While RE does not directly kill tumor cells, it temporarily increases membrane permeability, allowing the delivery of therapeutic agents such as drugs or genes—a process known as electrochemotherapy (ECT). This technique has shown promising results in various cancer types, particularly in the treatment of cutaneous and subcutaneous metastases (33–35). Furthermore, the integration of ECT with other treatment modalities, such as immunotherapy and gene therapy, is being explored to enhance its efficacy and broaden its application in oncology (36, 37). This also provides a theoretical possibility for the combination of RE with oncolytic viruses.

Oncolytic virus is an emerging therapeutic regimen able to selectively kill tumor cells and has achieved remarkable results in clinical applications and basic research for various types of cancers (16, 38–40). It is noteworthy that the oncolytic virus H101 has also achieved significant efficacy in inhibiting or treating tumor recurrence. A case report by Wang et al. demonstrated pathological complete response in a patient with postoperative lymph node metastasis of colorectal cancer following intratumoral injection of oncolytic virus H101 in combination with capecitabine therapy (41). Additionally, another study has shown that H101 exhibits favorable efficacy in the treatment of malignant ascites (42). However, there are relatively few reports on the use of H101 oncolytic virus in the treatment of pancreatic cancer. Given IRE’s ability to preserve tumor vasculature and enhance the permeability of cell membranes, we hypothesized that it could facilitate the penetration of H101 virus into tumor tissues, improving infection efficiency and cytotoxicity. Experiments confirmed that RE alone did not inhibit tumor growth but enhanced migration, while the H101 virus significantly suppressed tumor proliferation and migration. Combined treatment amplified these effects, reducing tumor growth more effectively than either approach alone. Immunohistochemical analysis revealed lower Ki-67 expression in the combination group, indicating reduced tumor cell proliferation.

To further elucidate the mechanism by which RE combined with H101 oncolytic virus kills tumor cells compared to RE alone, we collected tumor tissues from mice and performed bulk RNA-seq and scRNA-seq. The GO and KEGG enrichment analysis results from bulk RNA-seq presented the top 10 pathways in descending order of significance. The results indicated that both the MAPK and apoptosis pathways are significantly activated. This conclusion was further corroborated by Gene Set Enrichment Analysis (GSEA) and KEGG pathway analysis results from scRNA-seq. Subsequent protein analysis revealed that combined treatment specifically upregulated JNK phosphorylation, with no changes in ERK or p38. JNK, a key regulator of the MAPK pathway, plays a central role in apoptosis (43, 44). Flow cytometry confirmed increased apoptotic cell proportions in the combination group, while Western blot analysis showed elevated pro-apoptotic BAX and reduced anti-apoptotic Bcl-2 expression. It is noteworthy that with the addition of the JNK inhibitor (SP600125), the apoptotic effect of the combined treatment group was attenuated, and the expression changes of apoptosis-related proteins were significantly reversed. This clearly indicates that JNK plays a crucial and direct role in activating the intrinsic apoptotic pathway and initiating the intracellular death program in the combined treatment group.

This study has certain limitations. Due to the slow growth of mouse tumors and the extended experimental period, the number of tumor specimens meeting sequencing requirements was limited. In addition, the high cost of single-cell sequencing further limits the ability to perform large-scale sample analysis. As a result, there may be potential selection bias in the sample selection and comparison process. In addition, this study primarily focused on the cytotoxic effects of H101 on tumor cells following infection, without further investigating its replication ability within cells. This mechanism warrants further investigation in future studies to optimize the application of H101 oncolytic virus and enhance its clinical efficacy.

## 5 Conclusion

In conclusion, for tumor cells surviving IRE treatment due to uneven electric field distribution, the combined use of the H101 oncolytic virus offers a promising adjuvant strategy. This combination enhances therapeutic efficacy by activating the JNK-MAPK pathway, leading to increased tumor cell apoptosis. Further clinical studies are warranted to validate this approach and explore its potential in improving outcomes for pancreatic cancer patients.

## Data Availability

The data presented in the study are deposited in the CNGBdb repository, accession number CNP0007003.
